# Estradiol promotes rapid degradation of HER3 in ER-positive breast cancer cell line MCF-7

**DOI:** 10.1016/j.bbrep.2018.10.008

**Published:** 2018-10-26

**Authors:** Junko Suga, Keiko Izumiyama, Nobuyuki Tanaka, Shigehira Saji

**Affiliations:** a Department of Medical Oncology, Fukushima Medical University, 1 Hikariga-oka, Fukushima-shi, Fukushima 960-1295, Japan; b Division of Cancer Biology and Therapeutics, Miyagi Cancer Center Research Institute, 47-1 Nodayama, Medeshima-Shiode, Natori, Miyagi 981-1293, Japan; c Division of Cancer Immunobiology, Department of Cancer Medical Science, Tohoku University Graduate School of Medicine, 2-1 Seiryo Aobaku, Sendai 980-0875, Japan

**Keywords:** HER3, Nedd4-1, Estradiol, ER, Degradation

## Abstract

HER3, a member of the receptor tyrosine kinase super family, is overexpressed in a number of cancers, and is associated with malignant phenotypes. Control of the protein stability of the membrane, as well as nuclear receptors, has been known to be an important process affecting tumor cells; however, their relationships have yet to be elucidated. In this study, we demonstrate that estradiol promotes rapid degradation of HER3 via the proteasome pathway in ER-positive breast cancer, MCF-7. ER prevented HER3 degradation, and knockdown of ER expression by si-RNA promoted rapid degradation of HER3. Breakdown of HER3 and ER were regulated by a ubiquitin ligase Nedd4-1 in the presence of estradiol stimulation. We speculate that estradiol quickly degrades ER, making HER3 accessible by Nedd4-1, and leads to the rapid degradation of HER3. In addition, knockdown of ubiquitin ligase Nedd4-1 enhances estradiol induced cell proliferation. These results indicate that HER3 and Nedd4-1 in ER-positive breast cancers might be an important therapeutic target.

## Highlights

• First time we found that estradiol promotes rapid degradation of HER3 in ER-positive breast cancer MCF-7 cells.
• Knockdown of ubiquitin ligase Nedd4-1 decreased the degradation efficiency of HER3 in the presence of estradiol.
• Knockdown of Nedd4-1 enhanced estradiol induced tumor cell proliferation.

## 1 Introduction

HER3 is a member of the receptor tyrosine kinase family (RTK) and lacks intrinsic tyrosine kinase activity in the C-terminal tail. It is activated by Heregulin-1 (HRG-1) stimulation, and plays a regulatory role in cell proliferation and migration [1], [2], [3]. Overexpression of HER3 has been reported in breast, ovarian, pancreatic and gastric cancers, and is significantly associated with cancer malignancy [4], [5], [6], [7], [8], [9], [10], [11], [12], [13]. However, the mechanisms of HER3 overexpression are still not well understood. On the other hand, previous studies have reported that HER3 is regulated by ubiquitination and degradation with HRG-1 stimulation [14], [15], [16], [17].

Ubiquitination is controlled by regulatory proteins in the ubiquitin-conjugation system, and occurs through the three sequential classes of enzymes: ubiquitin-activating enzyme (E1), ubiquitin-conjugating enzyme (E2) and ubiquitin ligase (E3) [18], [19], [20]. Ubiquitin ligases interact physically with the substrate to determine the protein's fate by leading to degradation. Previous studies have reported that the HER family receptors are degraded with a help of their specific ubiquitin ligases [21], [22]. For example, HER1 is degraded by ubiquitin ligase c-Cbl [23], HER2 is mediated by c-Cbl [24], [25], [26] or chaperon-interacting protein (CHIP) [27], [28]. HER4 is ubiquitinated by WWP1 [32] or Itch [33]. In the case of HER3, HRG-1 stimulation leads to proteasome-mediated degradation of HER3, and at least three ubiquitin ligases, including neuregulin receptor degradation protein-1 (Nrdp1) [14], [15], [16], [17], [29], neural precursor cell expressed developmentally down-regulated 4 (Nedd4-1) [30] and Itchy (Itch) [31], have been identified in the degradation process. Nevertheless, the functional relationship between HER3 ubiquitination and hormones remains unknown.

In our attempt to investigate breast cancer, we have been exploring the biological role of estradiol in estrogen receptor (ER) positive breast cancer. In this line, we found that estradiol promotes rapid degradation of HER3 via the proteasome pathway, and an ubiquitin ligase Nedd4-1 controls this process. Furthermore, Nedd4-1 affects proliferation of MCF-7 cells through its dual action on HER3 and ER.

## 2 Materials and methods

### 2.1 Cell culture

Human embryonic kidney cells 293 T and human breast cancer cell lines MCF-7 and MDA-MB231 were purchased from ATCC, and human breast cancer cell lines SKBR3 and BT474 were gifted by Dr. S. Hayashi (Tohoku University, Miyagi, Japan). The cells were cultured in DMEM (Wako) or RPMI 1640 (Wako) supplemented with 10% heat-inactivated FBS (Biowest), 100 units/ml penicillin G and 100 µg/ml streptomycin. For experiments evaluating the effect of 17β-estradiol (estradiol, Sigma-Aldrich), the MCF-7 cells were cultured for two days in phenol red-free DMEM (PRF-DMEM, Wako) containing 10% heat-inactivated FBS stripped of steroids by absorption to dextran-coated charcoal (DCC-FBS, Biological Industries). The cells were then cultured in a humidified 5% CO_2_ incubator at 37 ℃.

### 2.2 Reagents and antibodies

The reagents used were as follows: epoxomicin (Peptide); ethanol, cycloheximide and chloroquine diphosphate (Wako); dimethyl sulfoxide and Fulvestrant (Sigma-Aldrich). The antibodies used were as follows: anti-HER3, anti-NEDD4-1 and corresponding secondary antibodies (Cell Signaling Technology); anti-Itch and anti-Nrdp1 (Santa Cruz Biothechonology); anti-ER (Thermo Scientific): anti- β actin (Sigma-Aldrich).

### 2.3 siRNA and shRNA mediated knockdown

Knockdown of human ER was performed using si-ER (Ambion, catalog# 4392420), along with a non-silencing control si-RNA (catalog# 4390843). MCF-7 cells were transiently transfected with 10 µM of the si-RNAs using Lipofectamine RNAiMAX Transfection Reagent (Life Technologies) according to the manufacturer's protocol, and were further cultured for 48 h before assays.

Human sh-Nedd4-1 expressing lentivirus vectors were constructed in pRSI12-U6-sh-HTS4-UbiC-TagRFP-2A-Puro plasmid (Cellecta). The pRSI12-U6-sh-Nedd4-1-HTS4-UbiC-TagRFP-2A-Puro or control pRSI12-U6-sh-HTS4-UbiC-TagRFP-2A-Puroplasmid was transfected into 293 T cells together with two packaging plasmids, pCMV-VSV-G/RSV-Rev and pCAG-HIVgp (RIKEN Bio-resource Center), using FuGENE HD (Promega) according to the manufacturer's protocol. At 48 h post-transfection, the supernatants were collected and filtrated through a 0.45 µm syringe filter. The lentiviral particles encoding the shRNA that targeted Nedd4-1 or a scramble control were incubated with MCF-7 cells for 48 h. Transduced cells were selected for additional incubation for 72 h in the presence of 1 µg/ml puromycin (Wako). Cells were used immediately after selection.

### 2.4 Cycloheximide chase assay

MCF-7 cells were plated in 6-well culture plates at a density of 4 × 10^5^ cells/well with PRF-DMEM containing 10% DCC-FBS. After overnight incubation, the medium was replaced with serum-starved PRF-DMEM for 1.5 h. The cells were then treated with 5 μM epoxomicin (Epx), or 1 μM chloroquine (CQ) with 50 µg/ml cycloheximide (CHX) for 30 min, and chased for different time periods in the presence or absence of estradiol with CHX.

For CHX chase assay using Fulvestrant (Ful), MCF-7 cells were cultured in PRF-DMEM containing 10% DCC-FBS for 2 days. The cells were then plated in 6-well culture plates at a density of 4 × 10^5^ cells/well, with PRF-DMEM containing 10% DCC-FBS added to DMSO as control or 0.1 μM Fulvestrant. After overnight incubation, the medium was replaced with serum-starved PRF-DMEM for 1.5 h in the presence of DMSO or Fulvestrant. The cells were then treated with 50 µg/ml CHX for 30 min and chased for different time periods in the presence or absence of estradiol with CHX.

The cells were collected at each time point and processed for immunoblotting by anti-HER3, anti-Nedd4-1, anti-ER and anti-β actin antibodies.

### 2.5 Western blotting

Cells were grown in PRF-DMEM containing 10% DCC-FBS in 6-well culture plates. The cultured cells were then washed twice with ice-cold PBS before they were lysed in RIPA buffer (40 mM Tris-HCl, pH7.5, 1% NP-40, 150 mM NaCl, 2 mM EDTA, 2 mM Na_3_VO_4_, 50 mM NaF) containing protease inhibitor cocktail (Roche). Lysates were scraped, transferred into microtubes, and centrifuged at 13,000 g for 20 min at 4 ℃. The supernatants were used as cell extracts. Total protein concentrations were determined using a Quick Start Bradford protein assay (Bio-Rad) using bovine serum albumin as a standard. Immunoblotting was subjected to 4–20% sodium dodecyl sulfate-polyacrylamide gel electrophoresis (SDS-PAGE, Bio-Rad), followed by transference to 0.45 µm pore size polyvinylidene difluoride membranes (Millipore), and blotting with primary and secondary antibodies. Quantification was performed using ImageJ software.

### 2.6 Cell proliferation assay

Cell proliferation was detected with a Cell Counting Kit-8 (CCK-8, Dojindo) according to the manufacturer's protocol. The sh-control or sh-Nedd4-1 knockdown MCF-7 cells were seeded in 96-well culture plates (7 × 10^3^ cells/well) in PRF-DMEM containing 10% DCC-FBS. After overnight incubation, the cells were replaced in a medium containing ethanol (EtOH) or 1 nM estradiol reagent. At 0, 24, 48, and 72 h of incubation, 10 µl of CCK-8 solution was added to the cells. After incubating the cells for 2 h at 37 ℃, absorbance at 450 nm was measured using a plate reader (Thermo Scientific).

### 2.7 Statistical analysis

All data are expressed as mean ± SD, as indicated in the figure legends. Statistical analysis was performed using the *Student t-test*. Significance is denoted as * , P < 0.05; * *, P < 0.01. All experiments were replicated at least three times.

## 3 Results

### 3.1 HER3, ER and Nedd4-1 express in MCF-7 cells

**Fig. 1:**
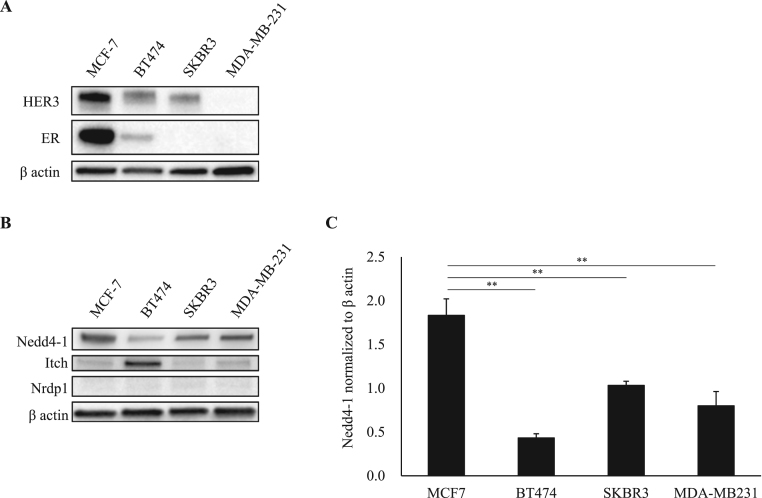
**Expression of HER3, ER and ubiquitin ligases in human breast cancer cell lines.** (A) Endogenous HER3, ER and (B) ubiquitin ligase (Nedd4-1, Itch and Nrdp1) expression in the four subtypes of human breast cancer cells (MCF7; Luminal A, BT474; Luminal B, SKBR3; HER2, MDA-MB231; triple negative) were analyzed by immunoblotting with anti-HER3, anti-ER, anti-Nedd4-1, anti-Itch, anti-Nrdp1 and anti-β actin antibodies. (C) Densitometry analysis of immunoblots. The quantification of the Nedd4-1 protein levels was done using ImageJ software. The protein levels were normalized to β actin levels. The results shown are from three independent experiments. * *P < 0.01 versus β actin. Error bars represent mean ± SD.

To confirm the expression of endogenous HER3 and ER in human breast cancer cell lines (MCF-7, BT474, SKBR3 and MDA-MD-231), we examined the Western blotting results. HER3 was expressed in the MCF-7, BT474 and SKBR3 cells (Fig. 1A, upper panel), and expression of endogenous ER was confirmed in the MCF-7 and BT474 cells (Fig. 1A, second panel). We next examined whether Nedd4-1, Itch and Nrdp1 ubiquitin ligases were expressed in the four breast cancer cell lines. Nedd4-1was highly expressed in the MCF-7 cells compared to that in the other cells (Fig. 1B, upper panel and Fig. 1C). Itch was remarkably detected in the BT474 cells (Fig. 1B, second panel). The endogenous Nrdp1 expression was very low in all of the breast cancer cell lines (Fig. 1B, third panel). From these results, we chose the MCF-7 cells for further study, since appreciable amounts of ER and HER3 were expressed in those cells.

### 3.2 HER3 is rapidly degraded in the presence of estradiol via proteasome pathway

To evaluate the degradation speed of HER3 in the presence or absence of estradiol, we performed the CHX chase assay, which monitors the amount that proteins decrease under the *de novo* protein biosynthesis inhibition with CHX. Ethanol (EtOH) was solvent of estradiol and was used as control stimulation.

**Fig. 2:**
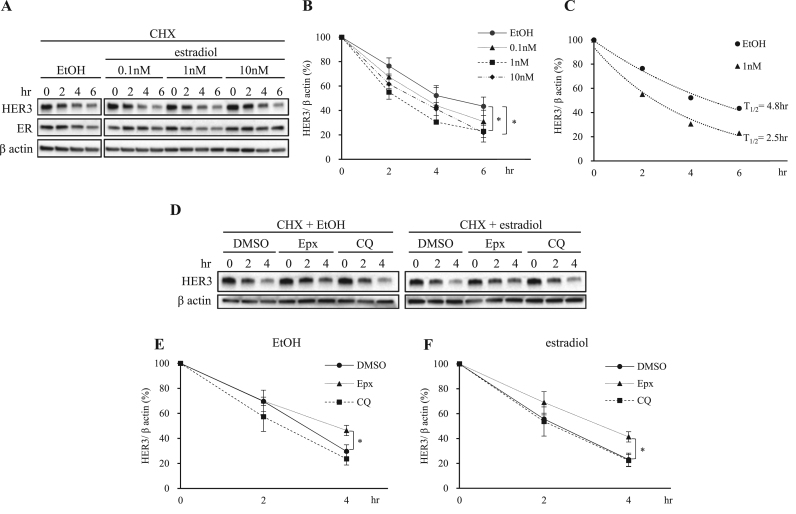
**Estradiol induces rapid degradation of HER3 via proteasome pathway.** (A) MCF-7 cells were incubated with serum-starved PRF-DMEM for 1.5 h. The cells were then treated with 50 µg/ml cycloheximide (CHX) for 30 min, followed by treatment with indicated concentrations of estradiol. The cells were lysed at indicated time points and subjected to immunoblotting for anti-HER3, anti-ER and anti-β actin antibodies. (B) The quantification of the HER3 protein levels was done using ImageJ software. The protein levels were normalized to β actin levels. The results are shown as means ± SD of three independent experiments. *P < 0.05 versus EtOH. (C) Half-life of HER3 was calculated based on the data in Fig. 2B. (D) The MCF-7 cells were incubated with serum-starved PRF-DMEM for 1.5 h. The cells were treated with 5 μM epoxomicin (Epx), 1 μM chloroquine (CQ), or DMSO with 50 µg/ml CHX for 30 min, followed by treatment with 1 nM estradiol or EtOH in the presence of CHX. The cells were then lysed at indicated time points and subjected to immunoblotting for anti-HER3 and anti-β actin antibodies. (E, F) Quantification of the HER3 protein levels was done using ImageJ software. Protein levels were normalized to β actin levels. All values are shown as means ± SD of three independent experiments. *P < 0.05 versus DMSO.

Among the several concentrations of estradiol tested, 1 nM estradiol induced the strongest HER3 degradation (Fig. 2A and B). Therefore, 1 nM estradiol seemed to be the most preferable concentration for evaluating the effect of estradiol on HER3 degradation (Fig. 2A and B, closed square). As shown in Fig. 2C, the half-life of HER3 shortened from 4.8 h to 2.5 h after 1 nM estradiol treatment. To identify the HER3 degradation pathway, we performed experiments using the proteasome inhibitor epoxomicin (Epx), or an endosome-lysosome system inhibitor chloroquine (CQ). In the presence of estradiol, Epx treatments, but not CQ, led to decreased HER3 degradation compared to the control treatment (DMSO), indicating that enhanced degradation of HER3 by estradiol depends on the proteasome pathway (Fig. 2D and F, closed triangle). In the absence of estradiol, Epx also prevented later degradation to some degree. This indicates that HER3 degradation is also mediated by the proteasome pathway (Fig. 2D and E, closed triangle). Higher degradation of HER3 with CQ treatment than with Epx treatment might be a secondary effect, likely due to the induction of another degradation process, although this remains to be confirmed (Fig. 2D and E, closed square). These results suggest that enhanced degradation of HER3 by estradiol is mediated through the proteasome pathway in MCF-7 cells.

### 3.3 Nedd4-1 regulates HER3 and ER degradation in the presence of estradiol

**Fig. 3:**
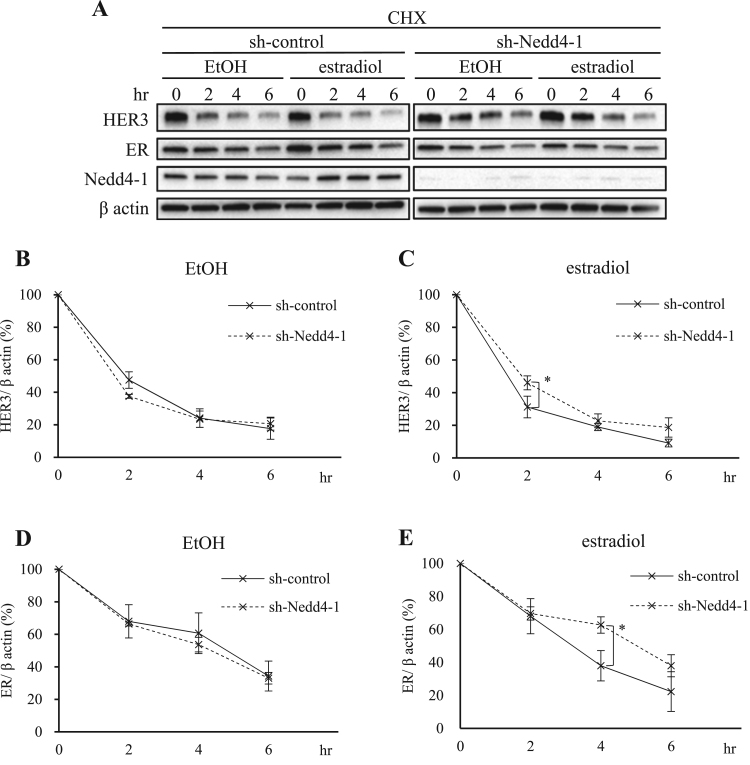
**Nedd4-1 regulates HER3 and ER degradation in the presence of estradiol.** (A) sh-control MCF-7 cells and sh-Nedd4-1 knockdown MCF-7 cells were incubated with serum-starved PRF-DMEM for 1.5 h. The cells were then treated with 50 µg/ml CHX for 30 min, followed by treatment with EtOH or 1 nM estradiol in the presence of CHX. All protein levels were assessed by immunoblotting at indicated time points. Quantification of the HER3 (B, C) and ER (D, E) protein levels were done using ImageJ software. The protein levels were normalized to β actin levels. All values are shown as means ± SD of three independent experiments. *P < 0.05 versus sh-control.

To determine whether Nedd4-1 contributes to the enhanced degradation of HER3 by estradiol, we established Nedd4-1 knockdown MCF-7 cells. Sh-control MCF-7 cells or sh-Nedd4-1 MCF-7 cells were treated with CHX at indicated time points with or without 1 nM estradiol. In the estradiol-stimulated condition, at the 2 h time point, HER3 degradation efficiency in the sh-Nedd4-1 MCF-7 cells (Fig. 3A and C, dotted line) was reduced to less than that in the sh-control MCF-7 cells (Fig. 3A and C, full line). In the absence of estradiol, no differences between the sh-Nedd4-1 MCF-7 (Fig. 3A and B, dotted line) and sh-control MCF-7 cells (Fig. 3A and B, full line) could be detected. This result indicates that Nedd4-1 plays a role in HER3 degradation under an estradiol-stimulated condition at a specific early time point, such as 2 h after stimulation.

To investigate the possible involvement of the same degradation process for ER, we used the CHX chase assay of ER proteins in the sh-control MCF-7 cells and sh-Nedd4-1 MCF-7 cells. After 4 h of estrogen stimulation, ER degradation in the Nedd4-1 knockdown MCF-7 cells (Fig. 3A and E, dotted line) was more suppressed than in the sh-control MCF-7 cells (Fig. 3A and E, full line). In the absence of estradiol, ER was similarly decreased in both the sh-control MCF-7 (Fig. 3A and D, full line) and sh-Nedd4-1 MCF-7 cells (Fig. 3A and D, dotted line). These results suggest that in the estradiol-stimulated condition, Nedd4-1 regulates the HER3 and ER degradation processes at a specific time point.

### 3.4 Depletion of ER promotes the rapid degradation of HER3

**Fig. 4:**
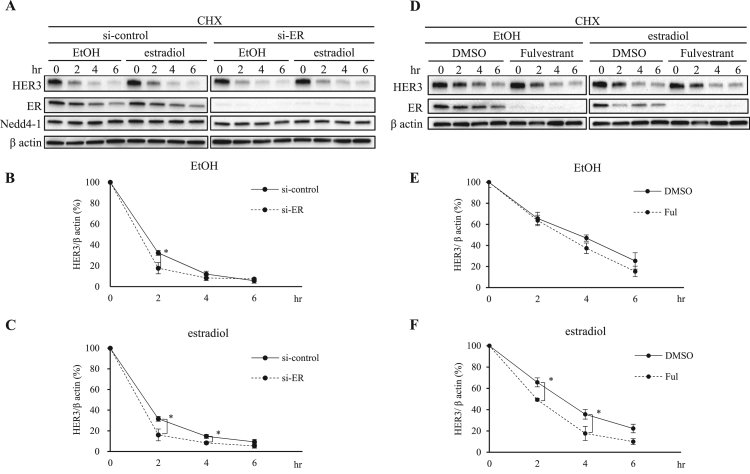
**Depletion of ER promotes the rapid degradation of HER3.** si-control MCF-7 cells and si-ER knockdown MCF-7 cells were incubated with serum-starved PRF-DMEM for 1.5 h. The cells were then treated with 50 µg/ml CHX for 30 min, followed by treatment with EtOH or 1 nM estradiol in the presence of CHX. All protein levels were assessed using immunoblotting at indicated time points. (B, C) Quantification of the HER3 protein levels was done using ImageJ software. All data from the three experiments were normalized to β actin. Mean values ± SD were plotted. *P < 0.05 versus si-control. (D) MCF-7 cells cultured in PRF-DMEM containing 10% DCC-FBS for 2 days. Cells were prepared with PRF-DMEM containing 10% DCC-FBS added to DMSO or 0.1 μM Fulvestrant. The medium was replaced with serum-starved PRF-DMEM for 1.5 h in the presence of DMSO or Fulvestrant. The cells were then treated with 50 µg/ml CHX for 30 min and chased for different time periods in the presence or absence of estradiol with CHX. The cells were collected at each time point and processed for immunoblotting by anti-HER3, anti-ER and anti-β actin antibodies. (E, F) Quantification of the HER3 protein levels was done using ImageJ software. The protein levels were normalized to β actin levels. All values are shown as means ± SD of three independent experiments. *P < 0.05 versus DMSO.*.

Since both HER3 and ER share the same ubiquitin ligase Nedd4-1 for their degradation under an estradiol-stimulated condition, we then examined whether the amount of ER affected HER3 degradation. We transiently suppressed ER expression using si-RNA. The ER knockdown MCF-7 cells (Fig. 4A and C, dotted line) promoted rapid degradation of HER3 compared to the si-control MCF-7 cells (Fig. 4A and C, full line) after 2–4 h treatment in the presence of estradiol. The same finding was also observed in the absence of estradiol, that ER itself protected HER3 from degradation (Fig. 4A-C).

To confirm our findings in the ER-knockdown study, we performed the CHX chase assay using Fulvestrant, a selective ER down-regulator, which degrades ER and acts as a complete antagonist to ER function. In the estradiol-stimulated condition, degradation of HER3 was remarkably enhanced for 2–4 h by pretreatment with Fulvestrant (Fig. 4D and F, dotted line). In the control condition (EtOH), pretreatment with Fulvestrant also enhanced HER3 degradation, but this difference was small and not significant. (Fig. 4. D and E, full and dotted lines).

From these results, we speculated that estradiol induces HER3 degradation, which then liberates HER3 from its inhibition by ER, eventually leading to the degradation of HER3.

### 3.5 Knockdown of Nedd4-1 enhances the proliferation of MCF-7 cells

**Fig. 5:**
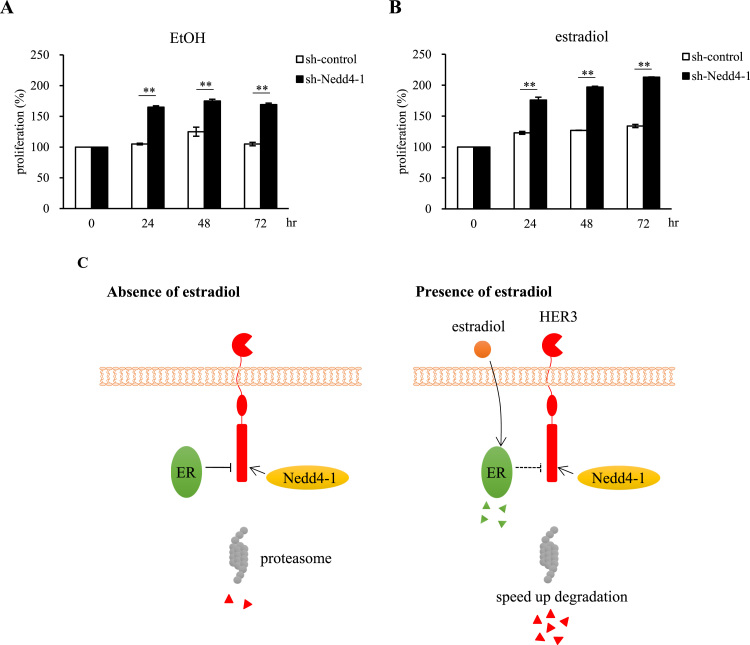
**Knockdown of Nedd4-1 enhances proliferation of MCF-7 cells.** (A, B) sh-control and sh-Nedd4-1 knockdown MCF-7 cells were incubated for the indicated time at 37 ℃ with EtOH or 1 nM estradiol. Cell proliferation was measured using CCK-8 assays. These data are representative of three independent experiments (n = 3). * *P < 0.01 versus sh-control. Error bars represent mean ± SD. (C) Summary of findings for the effect of HER3 degradation on ER-positive breast cancer cell line MCF-7.

To gain some insight into the biological role of Nedd4-1 in MCF-7 cells, we performed preliminary proliferation experiment using sh-Nedd4-1 MCF-7 cells. The Nedd4-1 knockdown in MCF-7 cells resulted in enhanced proliferation compared to the sh-control MCF-7 cells either with or without estradiol (Fig. 5A and B). These results suggest that Nedd4-1 may have a larger role in tumor biology, not only as a regulation molecule for ER and HER3 degradation upon estradiol stimulation, but also as an anti-proliferative factor in basic cancer biology.

## 4 Discussion

We showed here that estradiol promotes rapid degradation of HER3 in ER-positive breast cancer MCF-7 cells.

HER3 is a member of the HER family, which plays multiple roles in oncogenesis [34]. Overexpression of HER3 in several types of primary tumors or culture cells, such as that in breast, ovarian pancreatic and gastric cancers, has been reported [4], [5], [6], [7], [8], [9], [10], [11], [12], [13].

In breast cancer, HER3 contributes to tumor cell survival and proliferation, and previous reports have shown that HER3 in breast cancer cases is associated with poor prognostic factors, in terms of grade, lymph node metastasis and tumor size [5], [6]. Therefore, an underlying mechanism for HER3 overexpression might be a target for drug development for breast cancer. To this end, we used ER-positive breast cancer MCF-7 cells, which had remarkably positive HER3 expression compared to the cell lines that we evaluated (Fig. 1A).

In our HER3 CHX chase assay, we found that HER3 was degraded more rapidly in the presence of estradiol than in its absence (Fig. 2A and B). As is well-known, estradiol is a ligand of ER, and estradiol stimulation causes rapid ER degradation as a result of ligand-receptor interaction [35], [36], [37]. In the current study, the half-life of both ER and HER3 were affected by estradiol stimulation (Fig. 2A and C), leading to the suspicion that the same degradation mechanism was involved in both receptors. It is known that HER3 is quickly degraded by the proteasome pathway upon Heregulin-1 (HRG-1) stimulation and, interestingly, this is also true for estradiol stimulation, as shown in our proteasome pathway inhibitor experiments (Fig. 2D-F). Moreover, ubiquitination of HER3 was observed in the estradiol-stimulated condition (data not shown).

Degradation of HER3 under HRG-1 stimulation has been associated with three ubiquitin ligases, Nedd4-1, Itch and Nrdp1 [14], [15], [16], [17], [30], [31]. Nedd4-1 is the only ubiquitin ligase which was endogenously expressed in the MCF-7 cells, and it specifically contributed to the estradiol induced rapid degradation process of HER3 and ER. We suspect that Nedd4-1 is involved in the estradiol induced degradation of HER3 in a time dependent manner. In general, the degradation process is a time-dependent multi-step process, and involves various factors. In our study, we observed that the timing of Nedd4-1's contribution is different in ER (at 4 h) and HER3 degradation (at 2 h). This might be due to differences in interaction factors of Nedd4-1 in each degradation process (Fig. 3). Interestingly, depletion of ER enhanced HER3 degradation irrespective of estradiol stimulation (Fig. 4), indicating that ER might possess a function that prevents HER3 degradation through direct interaction. Collins et al. reported that HER3 forms a complex with ER in the presence and absence of HRG-1 [38], [39]. In the current study, we were unable to prove direct interaction between ER and HER3; however, we speculate that the formation of the ER/HER3 complex could prevent HER3 degradation. Together, our current hypothetical schema is shown in Fig. 5C. ER prevents HER3 degradation through its interaction under an estradiol-negative condition. Upon estradiol stimulation, ER is quickly degraded, and HER3, which is now free from ER, is led to prompt degradation. Richard et al. reported that growth factor receptors such as EGFR or HER2 cross-talk with ER and tend to acquire resistance to endocrine therapy [39]. Since HER3 and EGFR belong to same receptor family, we hypothesize that HER3 cross-communicates with ER. This ER-HER3 crosstalk would shed light on a previously unknown aspect of breast cancer research.

Finally, Nedd4-1 knockdown showed remarkable proliferative properties, both in the presence and absence of estradiol (Fig. 5A and B). Our observation that the enhanced effect of Nedd4-1 knockdown in HRG-1 stimulated proliferation is consistent with the results of previous report [30]. Nedd4-1 contributes to estradiol-induced proliferation of MCF-7 cells through interaction with HER3, although further investigation is needed to confirm this.

A limitation of our study is that we were unable to show the difference in the ubiquitination of HER3 between in the presence or absence of estradiol. The ubiquitination of HER3 seems to have occurred for a short time and we need to establish a more precise assay system. Although we performed several experiments for evaluating the PI3K/Akt and MAPK signaling pathways of HER3 to explain the biological impact of ER/HER3 interaction through a degradation process, we could not obtain significant results. This may be due to the experiment settings or incorrect target signaling.

In summary, our findings showed a model of estradiol-induced HER3 degradation in ER-positive breast cancer MCF-7 cells. HER3 was degraded rapidly by the proteasome pathway under estradiol stimulation, and ubiquitin ligase Nedd4-1 contributed to both HER3 degradation and tumor cell growth. The impact of Nedd4-1 on breast cancer biology should continue to be studied in future research.
