# Supplementary material for: Estradiol promotes rapid degradation of HER3 in ER-positive breast cancer cell line MCF-7
Source: Biochem Biophys Rep. 2018 Oct 26;16:103–9. doi: 10.1016/j.bbrep.2018.10.008 (PMC6205365; doi:10.1016/j.bbrep.2018.10.008)
Supplement: Supplementary file 1 — Supplementary material [file mmc1.docx]

Conflict of Interest:

Junko Suga has none to report.

Keiko Izumiyama has none to report.

Nobuyuki Tanaka reports a research grant from Boehringer-Ingelheim.

Shigehira Saji reports grants and lecture fees from Eisai, grants and lecture fees from Chugai, grants and lecture fees from Astra Zeneca, grants and lecture fees from Takeda, grants and lecture fees from Novartis, grants and lecture fees from Taiho, lecture fees from Kyowahakko Kirin, lecture fees from Pfizer, lecture fees from Daiichi Sankyo, grants and lecture fees from Nihon Kayaku, grants from Ono, outside the submitted work.

Other authors declare no conflict of interest.
